# The Influence of the Quality of Drinking Water in the Structure of the Teeth

**Published:** 1899-05

**Authors:** M. E. Louguet

**Affiliations:** Surgeon-Dentist of the Faculty of Paris


### The Influence of the Quality of Drinking Water in the Structure of the Teeth.

BY M. E. LOUGUET, SURGEON-DENTIST OF THE FACULTY OF PARIS.

Specially translated for the British Dental Journal.

In the issue of *L'Odontologie* for May 15th last, mention was made of the following statements contained in a work by Dr. Rose, of Munich: 1. The harder the water and the richer the soil in lime and magnesia, the better the structure of the teeth. 2. The softer the water and the poorer the soil in lime and magnesia the worse the teeth.

One naturally asks are these statements in accord with generally observed facts, and can definite conclusions be drawn from them? In the first place, it seems extraordinary to hold the view that water of high sp. gr. and therefore indigestible, should facilitate the nutritive changes which take place in the organism, and furthermore, it must be remembered that the lime necessary for building up the skeleton is not obtained from drinking-water alone, but also from the solid food on the nature of which much depends.

According to Dr. Rose, the inhabitants of districts having a calcareous subsoil should possess better teeth than those in countries where the subsoil is composed of primordial rocks, granite, crystalline schists, etc.

Now, according to Dr. Magitot, in the days when a faulty denture meant exemption from military service in France, the following differences per 100,000 conscripts were found to obtain: 108 in the West, South-West, and in the Central Plateau, against more than 1,100 in the East and the North. But to glance at the geological map of France shows at once that this does not tally with the nature of the subsoil. Certain other countries also tend to prove the inaccuracy of Dr. Rose's conclusions. Thus in Wales, Ireland, and a part of Scotland, where the older formations of soil vastly predominate, the teeth of the inhabitants are superior and less liable to decalcification than in England, which rests chiefly on calcareous formations.

There are two causes which appear to exercise considerable influence on the structure of the teeth and their power of resisting caries, viz., food and race. A flesh diet contains less of the salts necessary for calcification than a purely vegetable diet, and, moreover, the salts in the former are presented in a less assimilable form than the latter. But the nature of the food alone will not account for the differences in dental structure found in different regions. Here the racial element comes into play. Certain of the lower races (Australian aborigines), certain tribes of Central Africa, have a superior denture to that of the civilized white man, whilst from a dental point of view the Malays occupy an intermediate position between the negroes and the whites.—*L' Odontologie.*

